# Maximum Pain at Rest in Pediatric Patients Undergoing Elective Thoracic Surgery and the Predictors of Moderate-to-Severe Pain—Secondary Data Analysis

**DOI:** 10.3390/jcm13030844

**Published:** 2024-02-01

**Authors:** Lucyna Tomaszek, Dariusz Fenikowski, Nina Cież-Piekarczyk, Wioletta Mędrzycka-Dąbrowska

**Affiliations:** 1 Department of Thoracic Surgery, Institute of Tuberculosis and Lung Diseases, Rabka-Zdrój Branch, 34-700 Rabka-Zdrój, Poland; ltomaszek@igrabka.edu.pl (L.T.); dariusz.fenikowski@igrabka.edu.pl (D.F.); nina.ciez-piekarczyk@ppuz.edu.pl (N.C.-P.); 2 Department of Specialist Nursing, Faculty of Medicine and Health Sciences, Andrzej Frycz Modrzewski Krakow University, 30-705 Kraków, Poland; 3 Medical Institute, Academy of Applied Sciences in Nowy Targ, 34-400 Nowy Targ, Poland; 4 Department of Anaesthesiology and Intensive Care Nursing, Medical University of Gdansk, Gdans, 7 Debinki Street, 80-211 Gdansk, Poland

**Keywords:** multimodal analgesia, postoperative pain, moderate-to-severe pain, route of administration, predictors

## Abstract

Introduction: Pain management among children following thoracic surgery is an area of significant practice variability. Understanding the risk factors of moderate-to-severe pain intensity will allow for adequate pain relief. The aim of the study was to assess the maximum intensity of pain at rest in pediatric patients within 24 h of thoracic surgery and to investigate the prevalence and predictors of moderate-to-severe pain. Methods and findings: This is a prospective cohort study of patients in observational and randomized controlled trials following thoracic surgery. A secondary analysis of data was conducted using data collected from 446 patients aged 7–18 years undergoing thoracic surgery. The primary endpoint was maximum pain intensity (Numerical Rating Scale; NRS; range: 0–10) and the secondary endpoint was the prevalence and predictors of moderate-to-severe pain (NRS > 2/10). The median maximum pain in the cohort was 3 [0; 4]. During the immediate postoperative period, 54% of patients reported a maximum NRS > 2/10. The infusion of morphine by an intravenous route (vs. epidural route) was a protective factor against moderate-to-severe pain. Taking into account the findings related to the type of epidural analgesia (vs. intravenous morphine), it was found that only the administration of 0.25% bupivacaine combined with morphine or fentanyl was a protective factor against moderate-to-severe postoperative pain. Patients aged 14–18 years (vs. aged 7–13 years) had an increased risk of reporting pain as moderate-to-severe. Conclusions: The route of analgesic administration, type of multimodal analgesia, and patients’ age predict moderate-to-severe pain in pediatric patients after thoracic surgery.

## 1. Introduction

Thoracic diseases in pediatric patients necessitating surgical intervention include congenital lung malformations (e.g., lobar emphysema, pulmonary sequestrations), neoplastic or acquired lesions (e.g., thoracic trauma), diseases of infectious etiology (e.g., empyema thoracis), and chest deformities (e.g., pectus excavatum or carinatum) [1,2]. Surgery can be performed either via an open (e.g., thoracotomy, Ravitch procedure) [2,3], or a minimally invasive approach (e.g., thoracoscopic, Nuss procedure) [4,5].

Acute pain after thoracic surgery can caused by skin incisions, muscle splitting, intercostal nerve damage, pleural damage, chest drain insertion, retraction/resection/fracture of the ribs, or dislocation of the costovertebral joints [6]. Pediatric patients undergoing thoracic surgery show higher pain scores compared with those undergoing abdominal or extremity surgery [7]. Poorly treated pain in these patients can result, among other things, in atelectasis and pneumonia due to difficulty breathing deeply and coughing [6]. In addition, insufficient pain relief in the first 24 h after thoracic surgery is a risk factor for chronic pain [8].

Pediatric pain management after surgery with significant and extensive tissue damage is characterized by a multimodal approach. This involves combining smaller doses of opioid (e.g., morphine, fentanyl) and non-opioid analgesics (e.g., local anesthetics (LA), paracetamol, metamizole, nonsteroidal anti-inflammatory drugs (NSAIDs)), and other adjutants (e.g., gabapentin) to maximize pain relief and minimize drug-induced side effects [9,10]. Analgesics are given according to age-related pharmacokinetics. Intravenous opioid analgesia and thoracic epidural analgesia are commonly used in children undergoing non-cardiac thoracic surgery [11,12]. The thoracic paravertebral block is an alternative technique to epidural catheter in these patients. Furthermore, new methods of regional analgesia, such as intercostal nerve block and erector spinae plane block, are also applied as part of multimodal analgesia regimens [13,14,15].

According to the literature, pain in children after surgery peaks on the day of or the day after surgery [16,17]. The prevalence of moderate-to-severe pain (Numeric Rating Scale: 4 and above) in the early postoperative period varies significantly in different pediatric surgical specialties (25–76.7%) [17,18], and is very poorly understood in patients undergoing thoracic surgery [19]. The likelihood of having moderate-to-severe pain in the first 24 h of the postoperative period is higher among patients who suffer from preoperative anxiety and pain, and is related to the type of surgery and incision length [20]. The prognostic factors of postoperative pain may also include both male [21] and female gender [18], age [7], and analgesic technique. Current evidence suggests that the benefits of epidural analgesia are not as impressive as previously thought, but the risk of serious adverse events is greater than previously estimated [22].

However, the demographic, surgical, or analgesic factors that affect a patient’s pain postoperatively are still inconsistent and require further research. Therefore, the aim of this study was to assess the maximum intensity of pain at rest in pediatric patients within 24 h of thoracic surgery and to investigate the predictors of moderate-to-severe pain.

## 2. Materials and Methods

### 2.1. Study Design, Setting

**Figure 1:**
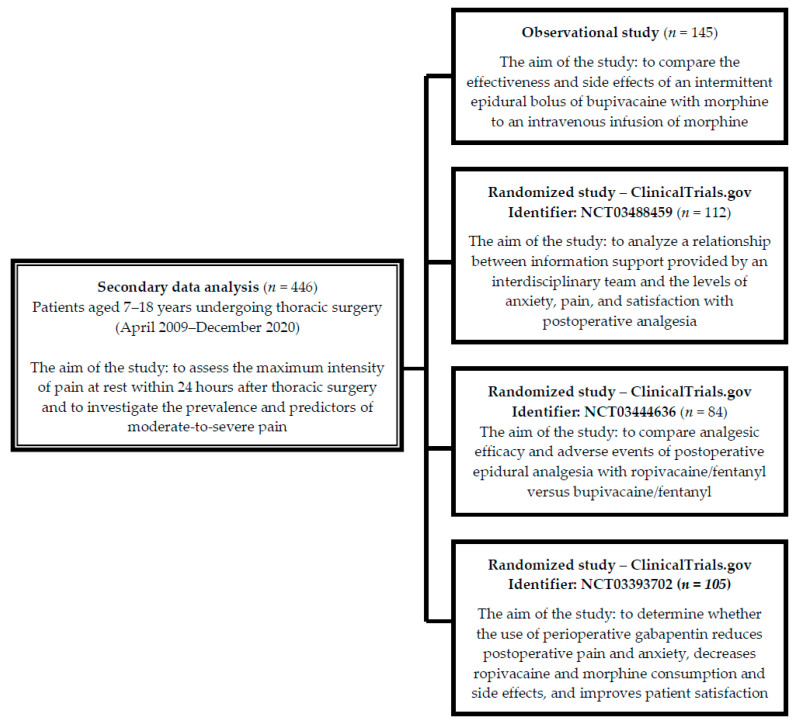
Flow diagram describing the secondary data analysis process.

This was a secondary data analysis of one observational trial [23] and three randomized controlled trials (Clinicaltrails.gov ID: NCT03488459, NCT03444636, and NCT03393702), with the primary aim of assessing the effect of postoperative pain management interventions. The above-mentioned trials included 446 pediatric patients, and were conducted between April 2009–December 2020 in the Department of Thoracic Surgery at the Institute of Tuberculosis and Lung Disease, Rabka Zdrój Branch, Poland (Figure 1).

### 2.2. Participants

Patients were considered eligible if they were 7 to 18 years of age, scheduled for elective thoracotomy or the Ravitch procedure, had postoperative chest drainage, were receiving postoperative analgesia via the epidural or intravenous route, were able to read and write in Polish, and were able to sign a consent form prior to the surgery (parents and patients aged ≥ 16 years).

Patients were excluded if they had allergies to any of the study drugs, were taking analgesics daily, were undergoing oncological treatment, or had a physical status > 3 according to the American Society of Anesthesiologists guidelines.

### 2.3. Postoperative Multimodal Analgesia

Intravenous analgesia consisted of the administration of morphine at a dose of 0.02–0.06 mg/kg per hour. An epidural infusion of 0.125–0.25% bupivacaine/0.2% ropivacaine with fentanyl 5 mcg/mL was given at a flow rate of 0.1 mL/kg per hour. On the other hand, 0.25% bupivacaine with morphine 0.1 mg/mL was administered by epidural catheter as a bolus every 8 h. Additionally, patients received weight-appropriate doses of non-opioid analgesics (paracetamol, non-steroidal anti-inflammatory drugs, and metamizol), either ‘as required’ (when pain occurs) or fixed scheduled ‘around the clock’ (irrespective of pain at the time of administration). Some patients were also given perioperative gabapentin. The detailed research methodology has been described in previous publications [23,24,25].

### 2.4. Data Collection

Demographic (age and gender) and clinical data were collected. The latter included: body weight and height, American Society of Anesthesiologists (ASA) classification, duration of surgery/anesthesia, type of surgery, chest drain, route of analgesic administration, type of postoperative multimodal analgesia, average and maximum pain at rest, and side effects experienced.

Pain within 24 h following thoracic surgery was recorded using the Numeric Rating Scale (NRS; range: 0 [no pain] to 10 [unimaginable pain]). A maximum NRS > 2/10 was defined as moderate-to-severe pain because in our previous study [26], 51.4% of children aged 7–18 years rated 3 as moderate pain. The NRS is strongly recommended as a self-assessment scale in this group of patients [27].

### 2.5. Outcomes

The primary outcome included the maximum intensity of pain. The secondary outcomes were the prevalence and predictors of moderate-to-severe pain (maximum NRS > 2/10).

### 2.6. Statistical Analysis

Continuous variables were summarized by presenting the median (upper and lower quartiles) and mean (standard deviation). For continuous data, intergroup differences were evaluated using Mann–Whitney and Kruskal–Wallis tests. The normality of data was tested using the Shapiro–Wilk test. The effect size, as a measure of clinical significance, was reported. The effect size was considered small, moderate, or large when Cohen’s d equaled or exceeded ±0.2, 0.5, or 0.8, respectively [28].

The criteria for Cohen’s effect size are guides rather than absolutes. Interpreting the response entails personal judgment vis-à-vis the practical or clinical importance of the effect. Variables that demonstrated a medium or greater effect were identified as being clinically meaningful, whereas outcomes with small effect sizes were identified as academically interesting but not clinically meaningful.

Categorical variables were summarized by presenting each category’s frequency and proportion of patients. Intergroup differences were assessed using the χ^2^ test or Fisher’s exact test.

The linear relationship between maximum pain and the number of non-opioid drugs was verified using the Spearman correlation coefficient (R), which was interpreted as negligible (<0.1), weak (0.1–0.39), moderate (0.4–0.69), strong (0.7–0.89), and very strong (0.9–1.0) [29].

A multiple logistic regression model was calculated to find the relationships between the dependent variable, “moderate-to-severe pain = NRS > 2” (Yes/No), and the independent variables, including age, gender, type of surgery, route of analgesia, type of analgesia, and gabapentin. Initially, a simple logistic analysis was carried out to select the predictors. A variable with a *p* value less than 0.1 was entered into the multiple regression model. An effective model was built using the backward elimination technique. The model was considered a good fit to the data as *p* > 0.05, according to the Hosmer–Lemeshow test. Nagelkerke’s R^2^ described the proportion of variance in the outcome that the model successfully explains. The significance of individual coefficients in the model were tested using Wald statistics. The odds ratio with a 95% confidence interval was also calculated.

All the calculations were made using STATISTICA v.13.3 (TIBCO Software Inc. (2017), Kraków, Poland). Values of *p* < 0.05 were considered significant for all the statistical analyses.

### 2.7. Ethics Consideration

The trials were previously approved by the Local Bioethics Committee at the National Institute of Tuberculosis and Lung Diseases in Warsaw, Poland (decision numbers: KE-40/2009, KB-64/2010, KB-2/2015, KB-6/2017, and KB-125/2019).

## 3. Results 

### 3.1. Patient Characteristics

**Figure 2:**
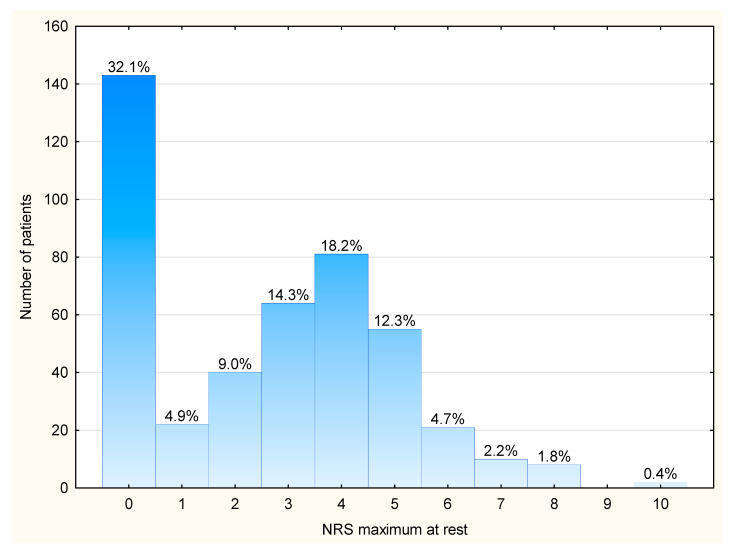
Histogram of the distribution of maximum pain intensity at rest in pediatric patients within 24 h of thoracic surgery. NRS = the Numeric Rating Scale; *n* = 446.

The study cohort consisted of 330 patients who underwent the Ravitch procedure (74%) and 116 patients who underwent a thoracotomy (26%). The median age was 14 years, the median weight was 52 kg, and the median height was 168 cm. The majority of patients were between 14–18 years of age (61%), male (76.4%), and had an ASA 1 physical condition (87.9%). The median duration of surgery and anesthesia was 140 and 190 min, respectively. Only 9.2% of patients had two chest drains and the rest had one drain. In 61% of patients, postoperative analgesia was administered by the epidural route, and 39% of patients received postoperative analgesia by the intravenous route. The median average pain intensity for the cohort was 0.4, whereas the median of maximum pain intensity was 3. Overall, 54% of the study cohort had an NRS > 2/10 (the percentage of severe pain (i.e., ≥7/10) was 4.4%). The baseline patient characteristics are presented in Table 1 and Figure 2.

### 3.2. Maximum Pain Intensity at Rest and the Prevalence of Moderate-to-Severe Pain (NRS > 2/10)

#### 3.2.1. Age

The prevalence of NRS > 2/10 was higher in patients aged 14–18 years than in younger patients (58% vs. 48%; χ^2^ = 4.22; *p* = 0.0398). However, there was no significant intergroup difference between the median pain scores (3 [0; 4] vs. 2 [0; 4]; Z = 0.85; *p* = 0.3903).

#### 3.2.2. Gender

The results of this trial revealed that there was no significant difference in pain intensity between female and male patients (median 3 [0; 5] vs. 3 [0; 4]; Z = −1.89; *p* = 0.0585). The incidence of moderate-to-severe pain was 59% and 52%, respectively (χ^2^ = 1.39; *p* = 0.2386).

#### 3.2.3. Type of Surgery

There were no significant differences between the thoracotomy and Ravitch procedure groups regarding median pain intensity (3 [0; 5] vs. 3 [0; 4]; Z = −1.89; *p* = 0.0586) and the prevalence of moderate-to-severe pain (57.8% vs. 52.7%; χ^2^ = 0.87; *p* = 0.3496). It should be noted that children had two drains more often after thoracotomy than after Ravitch surgery (27.6% vs. 2.7%; χ^2^ = 60.59; *p* < 0.00001).

#### 3.2.4. Route of Postoperative Analgesia

The median maximum pain for epidural and intravenous analgesia was 3 [0; 4], with means and standard deviations of 2.4 ± 2.2 and 2.9 ± 2.3, respectively. The *p* value for the Mann–Whitney test (Z = −1.99; *p* = 0.0457) indicates that there is a statistically significant difference in pain intensity between the groups, whereas Cohen’s d = 0.19, suggesting a small effect. An NRS > 2/10 was reported in 50.4% and 59.8% of patients, respectively (χ^2^ = 3.78; *p* = 0.0519).

#### 3.2.5. Type of Analgesia

**Figure 3:**
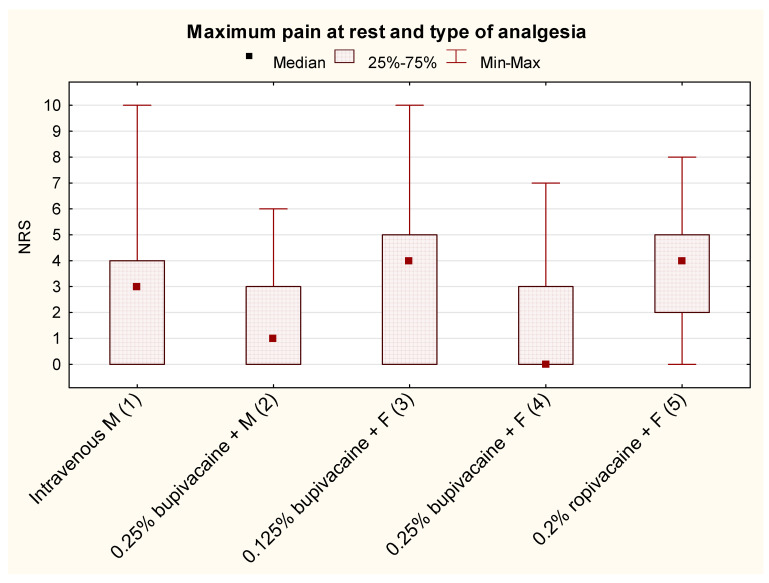
Maximum pain intensity at rest and the type of postoperative analgesia in pediatric patients within 24 h of thoracic surgery (M = morphine; F = fentanyl; Statistically significant intergroup differences—post hoc test results: 1 vs. 2, *p* = 0.0086; 1 vs. 4, *p* = 0.0001; 2 vs. 3, *p* = 0.0253; 2 vs. 5, *p* = 0.0002; 3 vs. 4, *p* = 0.0011; and 3 vs. 5, *p* = 0.000004).

The pain intensity results are shown in Figure 3. Among the analgesic mixtures administered by the epidural route, the infusion of 0.25% bupivacaine in combination with fentanyl was the most effective (median 0 [0; 3]). However, bupivacaine intake was approximately 3-fold higher compared to the 0.125% bupivacaine group (Table 2). Intravenous morphine was only less effective than 0.25% bupivacaine with morphine or fentanyl administered by the epidural route.

Statistically significant intergroup differences:

- Bupivacaine: 2 vs. 3 vs. 4, Kruskal–Wallis test: H (2, N = 190) = 109.47, *p* < 0.001; post hoc test results: *p* < 0.0001 for groups 2 vs. 4, and 3 vs. 4.
- Fentanyl: 2 vs. 3 vs. 4 vs. 5, Kruskal–Wallis test: H (2, N = 197) = 22.45, *p* < 0.0001; post hoc test results: 3 vs. 4, *p* = 0.0016; 4 vs. 5, *p* < 0.0001.
- Non-opioid: 1 vs. 2 vs. 3 vs. 4 vs. 5, Kruskal–Wallis test: H (4, N = 446) = 220.87, *p* < 0.001; post hoc test results: *p* < 0.0001 for groups 1 vs. 2, 1 vs. 3, 1 vs. 4, 1 vs. 5, 2 vs. 3, 2 vs. 5, 3 vs. 4, and 4 vs. 5.
- Itching, *p* = 0.0153.
- Oxygen saturation < 94%, *p* < 0.00001.

The number of doses of non-opioid drugs was weakly positively correlated with the intensity of maximum pain (R = 0.38; t = 8.72; *p* < 0.0001). Although patients aged 14–18 years received more doses than younger patients, there was no significant intergroup difference (median 6 [1; 7] vs. 4 [2; 7]; Z = 0.07; *p* = 0.9427). Among the non-opioid drugs, paracetamol was most frequently used (median 3 [1; 4]), followed by non-steroidal anti-inflammatory drugs (median 1.5 [0; 3]) and metamizole (median 0 [0; 1]).

The side effects of postoperative analgesia are shown in Table 2. A significant difference was detected among the groups regarding the incidence of itching (*p* = 0.0153) and oxygen saturation < 94% (*p* < 0.00001). It was observed that 13–17.3% of patients receiving bupivacaine + opioid epidural infusions experienced itching. The lowest incidence of oxygen saturation < 94% was in the group that received an intermittent epidural bolus of bupivacaine with morphine (8%), compared to those whose pain was treated by continuous infusion (34.5–59.3%). Moreover, in the epidural group (*n* = 272), 2.9% of cases of high sympathetic blockade (Anisocoria/Horner syndrome), 4.8% cases of paresthesia, and 1.1% cases of muscle trembling were observed. These symptoms disappeared spontaneously. In summary, postoperative analgesia in our patients was safe, regardless of the route and type of analgesic administered.

### 3.3. Predictors of Moderate-to-Severe Pain (Maximum NRS > 2/10)

Based on the results of multiple logistic regression, Table 3 shows the potential predictors of moderate-to-severe postoperative pain. The infusion of morphine by an intravenous route (vs. an epidural route) was a protective factor against moderate-to-severe pain. Taking into account the findings of the type of epidural analgesia (vs. intravenous morphine), it was found that only the administration of 0.25% bupivacaine combined with morphine or fentanyl was a protective factor against moderate-to-severe postoperative pain. On the other hand, patients aged 14–18 years (vs. patients aged 7–13 years) had an increased risk of reporting moderate-to-severe pain.

## 4. Discussion

This study revealed that a high proportion of patients reported moderate-to-severe maximal pain at rest after thoracic surgery. However, this does not mean that those patients were undertreated. The route of analgesic administration, type of multimodal analgesia, and patients’ age may predict moderate-to-severe pain.

The goal of the pain management strategy for our patients was to safely reduce their postoperative pain scores to an NRS ≤ 2/10. Although 54% of our patients had at least one episode of maximum NRS > 2 in the observational period, the average pain score below 1/10 suggested proper pain relief. Taking into account the average pain scores, it seems that patients in our cohort experienced greater pain relief compared with thoracotomy patients in the Mansfield et al. study [30] and adolescents after spinal fusion surgery in the Ocay et al. study [31]. In the early postoperative period, patients in the above-mentioned studies had pain scores of 3 or less. In the first study, patients were administered analgesics both by the epidural and non-epidural route (intravenous patient-controlled analgesia and other regional anesthetics), whereas in the second study, patients received intravenous PCA (patient-controlled analgesia).

### 4.1. Postoperative Pain and Route of Analgesia

This study aimed to establish the effectiveness of analgesia provided by an epidural or intravenous route in children and adolescents after thoracic surgery. The dominant method of treating postoperative pain in our cohort was, as in the Mansfield et al. study [30], epidural analgesia (61% and 77.3%, respectively). Their study detected a statistically significant difference in average pain scores between the thoracotomy epidural and no epidural groups, but only in the early postoperative period (1.7 ± 1.1 vs. 2.4 ± 1.5). In our study, we noted a similar relationship regarding maximum pain for epidural and intravenous routes (2.4 ± 2.2 and 2.9 ± 2.3), but the clinical effect was small. On the other hand, other authors concluded that thoracic epidural was just as effective as intravenous opioid PCA in decreasing early postoperative pain scores after thoracotomy for oncologic disease [32], minimally invasive video-assisted thoracoscopic surgery [33], and the minimally invasive repair of pectus excavatum [34].

### 4.2. Postoperative Pain and Type of Analgesia

This study also examined the differences between maximum pain scores depending on the type of postoperative analgesia, which included the intravenous infusion of morphine, the epidural infusion of bupivacaine/ropivacaine with fentanyl, and intermittent epidural bupivacaine with morphine. The multiple logistic regression analysis revealed that only the administration of intravenous morphine and 0.25% bupivacaine combined with morphine or fentanyl was a protective factor against moderate-to-severe postoperative pain.

We found that the analgesic effect of intravenous morphine was similar to that of an analgesic mixture consisting of 0.125% bupivacaine or 0.2% ropivacaine with the addition of fentanyl (5 mcg/mL). This finding supports the studies conducted by Cassady et al. [35] and O’Hara et al. [36], who found no differences in analgesic effectiveness, comparing a continuous epidural infusion of 0.1% and 0.125% bupivacaine with fentanyl (2.5 mcg/mL and 5 mcg/mL, respectively) and intravenous morphine PCA.

The findings of this current study clearly indicate that a 0.25% concentration of bupivacaine better relieved pain than 0.125% bupivacaine, 0.2% ropivacaine, or intravenous morphine. Winnie and colleagues [37] revealed in their meta-analysis that only a 0.25% concentration of bupivacaine and ropivacaine was optimal in lowering pain 24 h after abdominal surgery for rectus sheath block. The lower concentration was found to be ineffective.

### 4.3. Postoperative Pain and Age

One of the findings that emerged from this study was that patients aged 14–18 years had an increased risk of reporting postoperative pain as moderate-to-severe compared to younger patients (aged 7–13 years). This is in accordance with a German cohort study investigating, among other things, age differences in epidural analgesia in children. Schnabel et al. [7], as the authors of the above-mentioned study, found that school children reported significantly lower pain scores compared to adolescents (aged 12–18 years) [7]. Chow et al. [38] also suggested, based on the results of their systematic review and meta-analysis, that older pediatric patients report higher postoperative pain; however, the association between these two variables was weak. In turn, Mekonnen et al. [20] did not identify child age as a predictor of moderate-to-severe pain among Ethiopian patients.

### 4.4. Strengths of the Current Study

The main strength of the current study is the sample size. To the best of our knowledge, this is the largest study documenting a pain management strategy in patients aged 7–18 years undergoing thoracic surgery.

### 4.5. Limitations of the Current Study

The weakness of this study is that the datasets were obtained from a single center, and the results may not be applicable to all types of major surgery. The findings of this study are limited by the use of non-opioid analgesics either ‘as required’ (when pain occurs) or fixed scheduled ‘around the clock’ (irrespective of pain at the time of administration). It should also be noted that the secondary database may not contain the exact variables that best fit the purpose of this study.

## 5. Conclusions

The results of this study indicate that the continuous intravenous infusion of morphine and the epidural infusion of 0.25% bupivacaine combined with opioid (morphine 0.1 mg/mL, fentanyl 5 mcg/mL) were protective factors against moderate-to-severe pain in pediatric patients after thoracic surgery. On the other hand, patients aged 14–18 years had an increased risk of reporting pain as moderate-to-severe compared to younger patients.

### Implications for Clinical Practice

The findings of this study do not support strong recommendations for the use of epidural analgesia in children and adolescents undergoing thoracic surgery. Intravenous morphine may be a good alternative to local anesthetics with opioids administered by the epidural route. However, the optimal analgesic protocol after thoracic surgery has not been defined, and the level of evidence is still limited. Further studies are required to better understand these aspects.

## Figures and Tables

**Table 1:** Baseline characteristics (*n* = 446).

Variables	*n* (%)	Me (Q_25_; Q _75_)	M (SD)
Age (years)		14 [12; 16]	13.7 ± 2.6
7–13 years of age and maximum pain	175 (39.2)	2 [0; 4]	2.5 ± 2.3
14–18 years of age and maximum pain	271 (60.8)	3 [0; 4]	2.7 ± 2.2
Gender and maximum pain			
Female	105 (23.5)	3 [0; 5]	3.0 ± 2.4
Male	341 (76.4)	3 [0; 4]	2.5 ± 2.2
Body weight (kg)		52 [43; 59]	51 ± 13
Body height (cm)		168 [157; 176]	165 ± 16
ASA			
1	392 (87.9)		
2	51 (11.4)		
3	3 (0.7)		
Type of surgery and maximum pain			
Thoracotomy	116 (26.0)	3 [0; 5]	2.9 ± 2.4
Ravitch procedure	330 (74.0)	3 [0; 4]	2.5 ± 2.2
Chest drain and maximum pain			
One	405 (90.8)		
Two	41 (9.2)		
Duration of surgery (min)		140 [117; 169]	148 ± 48
Duration of anesthesia (min)		190 [165; 220]	198 ± 50
Route of analgesic administration and maximum pain			
Intravenous	174 (39)	3 [0; 4]	2.9 ± 2.3
Epidural	272 (61)	3 [0; 4]	2.4 ± 2.2
Type of postoperative multimodal analgesia and maximum pain			
Intravenous M; continuous infusion	174 (39.0)	3 [0; 4]	2.9 ± 2.3
Epidural 0.25% bupivacaine + M; bolus	75 (16.8)	1 [0; 3]	1.8 ± 1.8
Epidural 0.125% bupivacaine + F; continuous infusion	54 (12.1)	4 [0; 5]	3.1 ± 2.6
Epidural 0.25% bupivacaine + F; continuous infusion	61 (13.7)	0 [0; 3]	1.4 ± 1.9
Epidural 0.2% ropivacaine + F; continuous infusion	82 (18.4)	4 [2; 5]	3.4 2.1
Gabapentin	54 (12.1)		
Average pain intensity (for the whole cohort)		0.4 [0; 0.8]	0.7 ± 0.8
Maximum pain intensity (for the whole cohort)		3 [0; 4]	2.6 ± 2.3
Maximum NRS > 2/10 (for the whole cohort)	241 (54.0)		

Morphine = M; Fentanyl = F; Me (Q25; Q 75) = median (upper and lower quartile); M (SD) = mean (standard deviation).

**Table 2:** The prevalence of moderate-to-severe pain (NRS > 2/10), consumption analgesics, and side effects in pediatric patients within 24 h of thoracic surgery.

	Type of Analgesia				
Variables	Intravenous M (1)	0.25% Bupivacaine + M (2)	0.125% Bupivacaine + F (3)	0.25% Bupivacaine + F (4)	0.2% Ropivacaine + F (5)
	*n* = 174	*n* = 75	*n* = 54	*n* = 61	*n* = 82
NRS > 2/10 (%)	104 (59.8)	27 (36.0)	33 (61.1)	17 (27.9)	60 (73.2)
LA (mg/kg)	–	2.2 [2.1; 2.4]	2.4 [1.9; 2.9]	6.1 [4.6; 7.2]	3.8 [2.9; 4.5]
Opioid (µg/kg)	725 [568; 921]	89 [85; 96]	9.7 [7.6; 11.1]	12.3 [9.2; 13.8]	9.4 [7.2; 11.3]
Non-opioid (number of doses)	4 [2; 7]	1 [1; 2]	7 [7; 8]	2 [1; 3]	7.5 [7; 8]
Side effects (%)					
Nausea/vomiting	61 (35.1)	29 (38.7)	25 (46.3)	26 (42.6)	36 (43.9)
Itching	13 (7.5)	13 (17.3)	7 (13)	10 (16.3)	3 (3.7)
Urine retention	7 (4.0)	6 (8.0)	1 (1.8)	2 (3.3)	0 (0.0)
Oxygen saturation < 94%	60 (34.5)	6 (8.0)	32 (59.3)	31 (50.8)	34 (41.5)
Anisocoria/Horner	0 (0%)	0 (0%)	0 (0%)	0 (0%)	8 (9.7%)
Paresthesia	0 (0%)	6 (8.0)	0 (0%)	0 (0%)	7 (8.5%)
Muscle trembling	0 (0%)	3 (4.0)	0 (0%)	0 (0%)	0 (0%)

NRS = Numerical Rating Scale; M = morphine, F = fentanyl; LA = Local anesthetics.

**Table 3:** Predictors of moderate-to-severe pain (maximum NRS > 2/10).

Variables	B	SE (B)	Wald Test	*p* Value	OR (Cl 95%)
**Simple logistic regression**					
Aged 14–18 ^reference category: Aged 7–13^	0.20	0.10	4.21	0.0402	1.22 (1.01–1.48)
Male	−0.13	0.11	1.38	0.2393	0.87 (0.70–1.09)
Ravitch procedure ^reference category: Thoracotomy^	−0.10	0.11	0.87	0.3500	0.90 (0.73–1.12)
Gabapentin ^reference category: No^	0.40	0.16	6.36	0.0117	1.49 (1.09–2.02)
Intravenous route ^reference category: Epidural^	0.19	0.10	3.76	0.0524	1.21 (0.998–1.47)
Type of analgesia ^reference category: Intravenous MF^					
Epidural 0.25% bupivacaine + MF	−0.64	0.22	8.75	0.0031	0.53 (0.34–0.81)
Epidural 0.125% bupivacaine + FNT	0.39	0.24	2.54	0.1107	1.47 (0.91–2.37)
Epidural 0.25% bupivacaine + FNT	−1.02	0.25	16.90	0.0000	0.36 (0.22–0.59)
Epidural 0.2% ropivacaine + FNT	0.94	0.22	17.82	0.0000	2.55 (1.65–3.95)
**Multiple logistic regression model**					
Aged 14–18 ^reference category: Aged 7–13^	0.27	0.10	6.64	0.010	1.31 (1.07–1.60)
Intravenous route ^reference category: Epidural^	−2.40	0.56	18.35	0.000	0.09 (0.03–0.27)
Type of analgesia ^reference category: Intravenous MF^					
Epidural 0.25% bupivacaine + MF	−1.60	0.35	20.95	0.000	0.20 (0.10–0.40)
Epidural 0.25% bupivacaine + FNT	−2.04	0.38	28.30	0.000	0.13 (0.06–0.27)

B = regression coefficient; SE = standard error; OR = odds ratio; CI = confidence interval; multivariable logistic regression model: R^2^Nagelkerka = 0.14; Hosmer–Lemeshow = 3.1594, *p* = 0.6754.

## Data Availability

The data that support the findings of this study are available from the main author (L.T.) upon reasonable request.
